# Effects of Health Education of Diabetic Patient’s Knowledge at Diabetic Health Centers, Khartoum State, Sudan: 2007-2010

**DOI:** 10.5539/gjhs.v6n2p221

**Published:** 2014-02-14

**Authors:** Fathia Osman MakkiAwouda, Taha Ahmed Elmukashfi, Seed Ahmed Hag Al-Tom

**Affiliations:** 1 Department of Medical Nursing, Faculty of Nursing Sciences, University of Khartoum, Khartoum, Sudan; 2 Department of Community Medicine, Faculty of Medicine, University of Khartoum, Khartoum, Sudan; 3 Curriculum Department, Faculty of Education, University of Khartoum, Khartoum, Sudan

**Keywords:** health education, diabetic patients, Khartoum state, Sudan

## Abstract

**Background:**

Educating and training diabetic patients is necessary for controlling and improving their health.

**Methods:**

It was Quasi-experimental study design study. The study aimed to determine the effects of health education on the achievements of diabetic patients regarding control and improvement of their health status; at Diabetic Health Centers in Khartoum State, Sudan; 2007-2010. The target populations were diabetic patients, who attended the diabetic health centers to receive their treatment. Using simple random sampling 152 patients were selected (58 males and 94 females). Before and after comparison was done. Data was processed using SPSS and pair t-test was used to determine the effect of health education. P-value equal or less than 0.05 was considered statistically significant.

**Findings:**

Test for before and after comparison was found to be statistically significant (p<0.05) for diabetic patients. They gained more knowledge after the implementation of the program; particularly in the areas of the nature and signs and symptoms of the disease, signs and symptoms of hypo & hyperglycemia, causes and warning signs of foot problems, foot care, and importance of exercises.

**Conclusion and Recommendations:**

Health education of diabetic patients is crucial for control of diabetes. Capacity building of diabetic health centers, strengthening diabetic patients association, and more research to study the effect of health education on diabetic patients were needed.

## 1. Introduction

Diabetes mellitus is a chronic disease in which the body cannot properly use the energy it gets from food (Danish2011). Regarding prevalence of diabetes in Africa in 2011, 14.7 million adults were estimated to have diabetes with regional prevalence of 3.8%, the highest prevalence in Africa Region was in the island of Reunion (16.0%) followed by Seychelles (12.4%), Botswana (11.1%) and Gabon (10.6%) (Phyllis, 2012). The number of diabetic patients admitted to hospitals in different parts of Sudan was about 15193 in 2008, 23200 in 2009, and 25654 in 2010. Out of these numbers 550, 760 and 607 patients died due to diabetes in 2008, 2009, 2010 respectively (Sudan National Ministry of Health 2011). People with diabetes constituted 7% of all hospital admissions in Sudan (A.M. Ahmed & N.H. Ahmed 2001).

### 1.1 Management of Diabetes Composed of

(i) Control diet: Each diabetic patient should have a personal meal plan. The dietary guideline for diabetes has changed, offering more flexibility in food planning. As it was stated by Lewis et al (2007) the components of diet for diabetic patient can be as follows: - Carbohydrates 45%-65%; Proteins less than 10%; Fats 25%-35%. (ii) Exercise: Patient has to develop an exercise plan with physician and diabetes educator; e.g. walking 10-15 minutes three days a week and gradually build up (Weil, 2007). (iii) Blood glucose testing: Every diabetic patient is encouraged to have a glucose meter at home to test his blood glucose level two hours after meals to see if diet plan controls it or not. Blood glucose levels should be kept at 90-130mg/dl in the morning when fasting and less than 180mg/dl after meals (Basavanthappa, 2009). (iv) Medication: Whatever drug is taken as oral pills or insulin patient should know name of medication, dose, and when to take it. All these guidelines can be done if we are able to give patients correct information and skills (Lewis, Heitkemper, Dirksen, O’brien&Bucher, 2007). (v) Education: Diabetic patients must be knowledgeable about nutrition, medication effects and side effects, exercise, blood glucose monitoring techniques and medication adjustment (Lewis et al., 2007). Health education intervention is necessary in the Sudan so as to raise the awareness of patients about their condition and improved quality of life. Education is not just a part of the diabetic treatment; it’s the treatment (Soundarya & Mohan, 2004). Patients’ education has effects on blood glucose level, body weight, blood pressure and serum creatinine (Iftkharuddin, Ahmed, & Rahila, 2001; Steven et al., 1986).

Reorientation and motivation of health personnel towards patient education regarding diabetes to create awareness, delay the complications, improve nutrition, reduce cost, and lower the hospital admissions. (Soundarya & Mohan, 2004)

Foot care knowledge significantly improved with education (Valk, Kriegsman, & Assendelft, 2001).

In Sudan; diabetes and its complications are considered as a major health problem, but it is preventable if we can teach patient how to manage himself properly e.g. 50% - 75% of lower extremities amputations were performed on people with diabetes. As many as 50% of these amputations can be avoided if we were able to teach patients about foot care measures and practice. Therefore, increasing knowledge build skills and developing attitudes that lead to improve metabolic status, quality of life, reduction or prevention of complications and facilitations of responsibility of decision–making and self care of people with diabetes is an important role to be played (Smeltzer & Bare, 2004).

### 1.2 Problem Statement

Health education for diabetic patients attending to Diabetic Health Centers at Khartoum State, Sudan, will be performed in the period 2007 – 2009; in order to raise their knowledge and awareness regarding diabetes management

### 1.3 Justification

During researcher’s work in Khartoum Teaching Hospital, observed that most of patients with lower limbs amputation have diabetes. This amputation can be avoided if we were able to teach patients about foot care (Smeltzer & Bare, 2004).

With an exception of Jabir Abu Aliz as specialized diabetes health center, no health education is given to patients in a proper way. So it is necessary to concentrate on diabetes education.

### 1.4 General Objective

To determine the effect of health education of diabetic patients; attending Diabetic Health Centers in Khartoum State; Sudan; on their achievement as regard to pre and post test knowledge; 2007 -2010

### 1.5 Specific Objectives

1) To study the demographic characteristics of diabetic patients attending for treatment at Diabetic Health Centers in Khartoum State; Sudan: 2007 -2010.
2) To determine the level of knowledge of diabetic patients attending for treatment at Diabetic Health Centers in Khartoum State; Sudan; before and after implementation of the program; i.e, pre- and post-test of their knowledge regarding different aspects of diabetic health care: 2007 -2010.
3) To study the effect of health education on the achievement of diabetic patients knowledge at Diabetic Health Centers in Khartoum State; Sudan: 2007 -2010.

## 2. Methodology

### 2.1 Study Design

It was a quasi experimental study.

#### 2.1.1 Study Area

Khartoum State, Sudan. There are two working diabetic health centers in Khartoum State; these are: **(1) Jabir Abu Aliz**: It is the first specialized public diabetic health center in Sudan. It has a daily frequency of more than 250 patients. **(2) Soba University Hospital:** It has an outpatient diabetic clinic.

#### 2.1.2 Study Population

They are diabetic patients who attended the diabetic health centers (in Khartoum State, Sudan) for treatment, their total number were 35,600; where 600 for Soba University Hospital while 35000 for Jabir Abu Aliz Center.

#### 2.1.3 Sample Size

A total of 152 diabetic patients were selected proportionally from these two centers; where three from Soba University Hospital and 149 from Jabir Abu Aliz Center. We started patient’s selection in July and continued up to September 2009; where we selected every patient who agreed to participate in the study, until the sample size was completed. The sample size was calculated using the following formula:


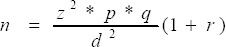


Where:

n =sample size

p =prevalence rate of diabetes = 0.1

q =non prevalence rate i.e. 1-p

z =standard value for 5% level of significance (z=1.96)

d=margin of desired error taken as 5%.

r =non response rate taken as 0.1

### 2.2 Tools of the Study

(i) Health education. (ii) Pre- and post tests for diabetic patients.

Components of health education program based on the pre and post test of diabetic patients and literature of educating and training of diabetic patients; consists of the following objectives: the content of the program, teaching methods and evaluation (International Diabetes Federation [IDF] 2008); (Smelter & Bare, 2004). So, we have: -

1) Simplified patho-physiological view: (a) Diabetes definition. (b) Normal range of blood glucose levels. (c) Exercise, food, stress and infections role. (e) Approaches of basic treatment.
2) Modalities of treatment: Insulin and oral hypoglycemic agent’s administration. (b) Information of diet. (c) Level of blood glucose and ketones monitoring.
3) Identifications, prevention and treatment of acute complications: (a) Hypoglycemia. (b) Hyperglycemia
4) Pragmatic information: (a) Source and storage of insulin syringes and monitoring of glucose supplies. (b) Map road to physician

### 2.3 Teaching Methods

Due to differences in the arrival time of patients to clinic; one - to one education was used.

### 2.4 Ethical Consideration

Technical and ethical approval of the program was obtained from the concerned committee.

Participant was fully informed about the study and then consent was obtained.

### 2.5 Pre- and Post Tests

A structured, pre tested questionnaire, composed of 31 questions, which covered demographic data and all aspects of the diabetes mellitus was used for assessment before and after the implementation of the health education program.

The total number of the diabetic patients attended the pre test was 152. These patients were taught and trained by diabetes health educators for three months. The time between intervention and posttest was three weeks. Then a post test was carried out for all educated patients (152).

### 2.6 Methods of Analysis

Two tests were used: Person correlation coefficient and Spearman and Brown equations to find out the validity and reliability of the two tests.

The equation used is:


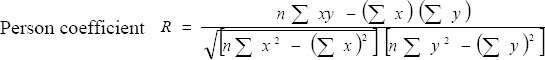



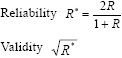


Where

n = sample size

R = Person coefficient

x = data for pretest

y = Data for posttest

The validity and reliability were 0.88 and 0.77 respectively.

### 2.7 Statistical Analysis

Data was processed using SPSS and pair t-test for one group was used.

## 3. Results and Discussion

Male and female composition of participants was (38.2%, 61.8%) respectively; most of them have low educational level, about 60.6% (illiterate, Quran school, primary); and 56.6% in the age group between 40-59 years.

As shown in table (1) the average knowledge for the nature of diabetes was 0.9408 before the implementation of the program and was raised to 1.74 after the implementation; the result was statistically significant with T-value 7.38 and P. value 0.000. These agree with many studies done by Soundarya & Mohan (2004) in India which found that education raised awareness about the disease, decreased the cost, and delay complications. For signs & symptoms of diabetes were 3.1854 and after the implementation was raised to 4.68; the result is statistically significant with T-value equals 8.90 and the P.value 0.000. The average patient’s knowledge of normal range of blood glucose level before the implementation of the program was 0.9605 and after the implementation was raised to1.50; the result is statistically significant with T- test value 4.57 and the P. value 0.000. Regarding signs & symptoms of hypoglycemia was 2.2763 before and 3.75 after the implementation; the result is statistically significant with T- test value 8.28 and P. value 0.000. The average knowledge for signs & symptoms of hyperglycemia was 1.7632 before and 3.11 after the implementation; the result is statistically significant with T- test value 7.55 and P. value 0.000.

**Table 1.** Average knowledge about nature, signs and symptoms of diabetes; normal range of blood glucose level; signs and symptoms of hypo- and hyperglycemia; before and after implementation of health education program among diabetic patients attending at Diabetic Health Centers in Khartoum State, Sudan; 2007 - 2010 (n=152).

Item	Assessment	N	Average	St. Dev	T-Value	P-Value
Nature of Diabetes	Before	152	0.9408	0.7116	7.38	0.000

	After	152	1.74	1.23		
Signs and symptoms of	Before	152	3.1854	1.4348	8.90	0.000
diabetes	After	152	4.68	1.75		
Normal range of blood	Before	152	0.9605	1.1210	4.57	0.000
glucose levels	After	152	1.50	1.28		
Signs and symptoms of	Before	152	2.2763	1.5955	8.28	0.000
hypoglycemia	After	152	3.75	1.64		
Signs and symptoms of	Before	152	1.7632	1.3007	7.55	0.000
hyperglycemia	After	152	3.11	1.95		

This assessment was done after three weeks post intervention.

As stated in Table 2 the average patient’s knowledge before and after implementation of the health education program was as follows: (i) Treatment of diabetes was 1.2105 and 1.78; the difference is statistically significant (P< 0.05); the result is statistically significant with T-valve 9.39 and P-value 0.000; there is a change before and after the implementation of the health education program but still below the benchmark; due to the diffusion of the knowledge. (ii) Self administer of insulin was 1.0789 and 2.27; result is statistically non significant with T- test value 1.95 and the P-value 0.053, but there is little improvement, most of patients on oral hypoglycemic agents and diet, also such type of activity needs practical and demonstration in addition to the theoretical lessons, so that they can gain knowledge, but the diabetic centers are not equipped for such activities. (iii) Side effects of treatment were 0.5724 and 0.86; result is statistically significant with T- test value 5.69 and the P-value 0.000. (iv) Dietary control was 0.6797 and 0.94; the result is statistically significant with T- test value 4.35 and the P- value 0.000; the result shows that patients know well about importance of diet for diabetics. (v) Importance of daily exercise was 1.14079 and 2.41; result is statistically significant with T- test value7.8 9 and P-value 0.000. (vi) Danger of strenuous exercises was 0.8553 and 0.67; result is statistically significant with T- test value 2.46 and P-value 0.015. (vii) Precautions to avoid side effects of strenuous exercises were 0.9211 and 1.52; the result is statistically significant with T- test value 5.27 and P-value 0.000. These findings indicate marked improvement in patient’s knowledge due to health education which may affect positively their health status.

**Table 2.** Average patient’s knowledge about: treatment of diabetes; self - administer insulin; side effects of treatment; importance of dietary control; importance of daily exercise; danger of strenuous exercises; and precautions to avoid side effects of strenuous exercises; before and after implementation of health education among diabetic patients attending at Diabetic Health Centers in Khartoum State, Sudan; 2007 - 2010 (n=152)

Item	Assessment	N	Average	StDev	T-value	P-value
Treatment of diabetes	Before	152	1.2105	0.5353	9.39	0.000
	After	152	1.78	0.59		
Self- administering of insulin	Before	152	1.0789	1.8999	1.95	0.053
	After	152	1.54	2.27		
Side effects of treatment	Before	152	0.5724	0.4964	5.69	0.000
	After	152	0.86	0.37		
Importance of dietary Control	Before	152	0.7697	0.4224	4.35	0.000
	After	152	0.94	0.24		
Importance of daily exercises	Before	152	1.4079	1.2145	7.89	0.000
	After	152	2.41	1.29		
Danger of strenuous exercises	Before	152	0.8553	0.7316	2.46	0.015
	After	152	0.67	0.63		
Precautions to avoid side	Before	152	0.9211	1.0455	5.27	0.000
effects of strenuous exercises	After	152	1.52	1.14		

From Table 3 we noticed that the average patient’s knowledge before and after implementation of health education program, regarding: (i) Causes of foot problems was 1.1819 and 1.57; the result is statistically significant with T- test value 7.38 and P-value 0.000. Such result agrees with study done by Valk, Kriegsman & Assendelft (2001), who found that education prevents foot ulceration and reduces incidence of disease and amputations. (ii) Warning signs of foot problems was 1.3 882 and 2.69; result is statistically significant with T- test value 4.90 and P-value 0.000. This indicates that if the patients able to do proper foot care they can detect warning signs early; so can be treated without complications. (iii) Foot care was 1.0329 and 2.37; the result is statistically significant with T- test value 8.08 and P-value 0.000.

**Table 3.** Average patient’s knowledge about causes of foot problems; warning signs of foot problems; foot care before and after implementation of health education among diabetic patients attending at Diabetic Health Centers in Khartoum State, Sudan; 2007 - 2010 (n=152)

Item	Assessment	N	Average	StDev	T-Value	P-value
Causes of foot	Before	152	1.2303	1.1819	7.38	0.000

problems	After	152	2.34	1.57	4.90	0.000
Warning signs of foot	Before	152	1.3882	1.3374		
problems	After	152	2.19	1.69		
Foot care	Before	152	1.0329	1.3143	8.08		0.000
	After	152	2.37	1.61			

## 4. Conclusion and Recommendations

Health education improved diabetic patient’s knowledge and raised their awareness, this is shown by the fact that most of the variables in post test are with significant value. Health education is suitable for different levels of age, sex and education. The suitable method for implementing heath education to diabetic patients is one to one; because they came at different times. The followings were recommended: Develop and equip more diabetic health centers with audio tapes, video tapes, pamphlets, leaflets, magazines, and books; strengthening of diabetic patients association; more research on evaluation of the impact of health education on diabetic patients; education should not be as a part of the treatment, it’s the treatment by organizing education session in diabetic health centers, and making an annual plan for each patient for his/her education; accessibility to diabetic health center should be easy for all diabetic patients and raising the awareness of diabetic patients towards their education.
